# Research Progress on Regulatory B Cells in Systemic Lupus Erythematosus

**DOI:** 10.1155/2019/7948687

**Published:** 2019-05-22

**Authors:** Tao Wang, Yongjun Mei, Zhijun Li

**Affiliations:** Department of Rheumatology, The First Affiliated Hospital of Bengbu Medical College, Bengbu 233004, China

## Abstract

Systemic lupus erythematosus (SLE) is a chronic, systemic, autoimmune inflammatory disease characterized by the production of numerous autoantibodies and cytokines, as well as multiple organ damage. Specific B cell subsets negatively regulate immune responses and have been termed regulatory B cells (Bregs). Bregs are characterized by the production of the immunoregulatory cytokines interleukin (IL)-10, IL-35, and transforming growth factor (TGF)-*β*. Bregs suppress other immune cells through the secretion of these immunosuppressive cytokines and have thus been studied extensively for their potential role in the treatment of various autoimmune diseases. The progress of the research on Bregs and SLE in recent years is reviewed in this paper.

## 1. Introduction

In general, B lymphocytes are thought to have a positive regulatory effect on humoral immune responses as they eventually differentiate into plasma cells and produce antigen-specific antibodies. However, a subset of B cells can negatively regulate the immune response and are named regulatory B cells (Bregs) [1]. There is evidence [1, 2] that Bregs play an important role in many mouse models of inflammation and autoimmunity. Although the definition of Bregs and their mechanism of action have only recently begun to be studied, it is undeniable as an important component of the immune system. Bregs have multiple phenotypes, but interleukin (IL)-10-producing Bregs are currently the most studied.

Systemic lupus erythematosus (SLE) is a systemic autoimmune disease characterized by a large amount of autoantibody production and deposition of immune complexes. Its clinical manifestations include multiple organ damage, including lesions in the skin and kidneys. An imbalance in type 1 (Th1) and type 2 (Th2) helper T cells, changes in the expression of related cytokines, and overactivation of Th2 cells are involved in the progression of SLE. As the main effector of Th2 cells, IL-10 participates in the activation and apoptosis of B lymphocytes, the regulation of antigen presentation, inflammation, and autoantibody production. In addition to directly participating in the proliferation and differentiation of B cells, IL-10 also indirectly affects the proliferation, survival, and secretion of B cells by stimulating the expression of B lymphocyte factor genes. This article will review the research progress on the Bregs involved in human SLE.

## 2. Definition and Phenotype of Bregs

The definition of human Bregs is primarily based on the production of IL-10, which is also heterogeneous in phenotype and function like that in mice [3, 4]. An overview of the different subsets that have been published in studies on humans is shown in Table 1. Human Bregs are categorized mainly as either transitional (CD19^+^CD24^high^ CD38^high^) or memory (CD24^high^CD27^+^) [3, 4]. CD19^+^CD24^high^ CD38^high^ B cells inhibit the production of proinflammatory factors by CD4^+^ T cells, relying on IL-10, CD80, and CD86, but not TGF-*β* [4]. CD24^high^CD27^+^ B cells produce IL-10 to regulate the production of TNF-*α* by monocytes [3]. CD19^+^CD25^high^ CD86^high^CD1d^high^ B cells also inhibit CD4^+^ T cell proliferation and increase the expression of FoxP3 and T lymphocyte antigen 4 in regulatory T cells by producing IL-10 and TGF-*β* [5]. In addition, IL-27 is increased in CD27^high^CD38^high^ plasmablasts [6]. Therefore, human Bregs are not of a single phenotype, but their regulation depends on IL-10 regardless of phenotype. Although there have only been a few studies on human Bregs, evidence suggests that they may become targets for the treatment of human diseases in the future.

The proportion of Bregs in human peripheral blood cells is usually less than 1% [4]. Human Bregs are generally considered to have a phenotype of either CD24^high^ CD27^+^ [4] or CD19^+^CD24^high^CD38^high^ [3]. The proportion of peripheral blood Bregs in SLE patients is higher than that in normal controls [4]. However, CD19^+^CD24^high^CD38^high^ B cells isolated from peripheral blood of SLE patients have a low response to CD40 stimulation, a lower production of IL-10, and a reduced inhibitory capacity [3].

Autoimmune diseases are caused by the immune system losing tolerance to autologous components, thereby initiating damage to autologous tissues and organs. Many studies have shown that inflammatory cytokines play an important role in the pathogenesis of autoimmune diseases. Recently, there has been increasing evidence that IL-10 is involved in the development and progression of autoimmune diseases. IL-10 affects the immune system and other pathophysiological processes by regulating cytokines and growth factors. IL-10 can increase peripheral tolerance of regulatory T cells and inhibit the secretion of cytokines by Th1 cells. IL-10 is also involved in the activation and apoptosis of B cells, the regulation of antigen presentation, and the regulation of inflammation and autoantibody production. In addition, the gene expression, the receptor structure, the associated gene polymorphisms, and the signaling pathway of IL-10 are all associated with the development of autoimmune diseases.

## 3. Bregs Participate in Human SLE through IL-10, IL-35, or TGF-*β*

**Figure 1:**
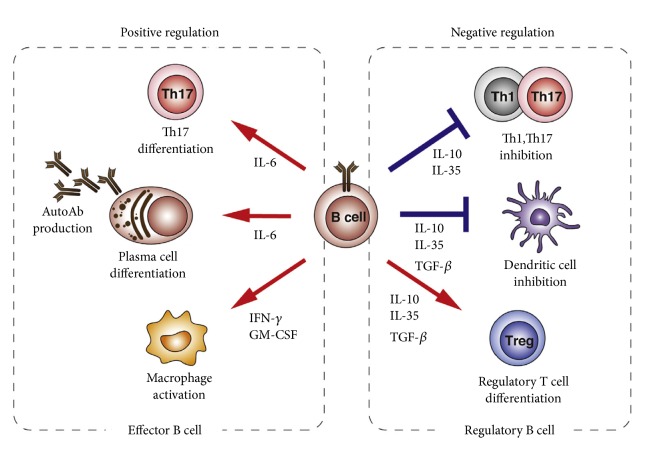
Regulatory and effector B cell function. Effector B cells positive regulate immune responses through provision of IL-6, IFN-*γ*, and GM CSF, while regulatory B cells negatively regulate immune responses through provision of IL-10, IL-35, and TGF-*β*.

The bidirectional regulation of B cells in autoimmune diseases includes functional and regulatory effects (Figure 1[1]). The balance between regulatory and effector functions is a finely regulated immunological process that is not fully understood.

In SLE, B cells represent an important group of effector cells whose function seems to depend on the presence of Bregs. The first clinical study of peripheral blood B cells in patients with SLE showed that the proportion of CD5^+^ B cells producing IL-10 was significantly higher than that in normal controls [16]. Another study showed that IL-10 production is closely related to the expression of CD154 on the B cell surface, suggesting the importance of cell activation for IL-10 production. Furthermore, IL-10-producing CD154^+^ B cells were increased after infection with Staphylococcus aureus Cowan 1 (SAC) in SLE patients [17].

Consistent with the above findings, Iwata et al. [3] also reported that IL-10-producing B cells were significantly increased in autoimmune disease, such as SLE, rheumatoid arthritis, sjogren syndrome, autoimmune bullous disease, and multiple sclerosis, when stimulated with lipopolysaccharide (LPS) or CpG (in combination with PMA and ionomycin, PI) for 48 hours with the involvement of CD40 ligand. Yang et al. [18] also found that CD19^+^IL-10^+^ B cells increased after 24 hours of LPS stimulation with PI in SLE patients. Recently, a study found that B cells are dysfunctional in autoimmune diseases. For example, SLE patients present with a functional decline in their CD24^high^CD38^high^ Bregs [4]. CD19^+^CD24^high^CD38^high^ Bregs inhibit the production of IFN-*γ* and TNF-*α* by CD4^+^ T cells after CD40 stimulation. In SLE patients, this inhibition does not occur, which may be attributed to IL-10. CD24^high^CD38^high^ Bregs isolated from peripheral blood of SLE patients did not respond to CD40 stimulation and produced less IL-10 than those from normal controls. Further studies [19] showed that, in SLE patients, the inhibition of T cell proliferation by B cells stimulated by IL-2 and SAC for 72 hours was weakened compared to normal controls. A recent study [20] also found that human type 3 innate lymphoid cells provide innate B cell help and are involved in an innate immunoregulatory mechanism involving the induction of immature transitional Bregs differentiation, which takes place in palatine tonsils in vivo. This mechanism, which can contribute to the maintenance of immune tolerance, becomes insufficient in allergic diseases. In conclusion, Bregs are more abundant in peripheral blood but are less functional.

A study [21] showed no difference in the proportion of CD19^+^ CD24^high^CD38^high^ cells in the peripheral blood of SLE patients and healthy controls. However, Yang et al. [18] defined the phenotype of Bregs as CD19^+^CD5^+^CD1d^high^, which are abundant in active SLE patients. Similar results were found in the study by Blair et al. [4], wherein the peripheral blood of patients with active SLE had a higher proportion of CD19^+^CD24^high^CD38^high^ cells than that of normal controls, but there was no difference in absolute numbers. However, a study [22] has also shown that the number of CD19^+^CD5^+^ B cells in peripheral blood of patients with SLE is decreased, while the level of IL-10 is increased. As the degree of disease activity increases, the changes in peripheral blood CD19^+^CD5^+^ B cells and IL-10 are more pronounced and can thus be used as an indicator to evaluate the activity of SLE.

Heinemann et al. [23] found that peripheral blood CD19^+^CD24^high^ CD38^high^ cells were not significantly different between the SLE and normal control groups. The IL-10^+^ Bregs in patients with SLE decreased compared with those in normal subjects, especially in SLE patients with lupus nephritis. However, there was no significant correlation between the proportion and the disease activity.

Mauri et al. [24] showed that plasmacytoid dendritic cells (pDC) regulate the differentiation of immature B cells into Bregs by producing IFN-*α*, thereby limiting the progression of inflammation. pDCs induces the differentiation of Bregs via an IFN-*α*-dependent pattern. Bregs limit the production of IFN-*α* by pDCs via an IL-10-dependent mechanism. pDCs are highly activated in SLE patients and do not induce differentiation of Bregs. In SLE patients receiving Rituximab monoclonal antibody, the relationship between pDCs and Bregs is normal.

Sieber et al. [25] observed that the decrease in IL-10 production was due to stimulation of Toll-like receptor 9 (TLR9). In SLE patients, the conclusions of the relevant studies on the production of IL-10 cells are different, mainly due to the different methods used in these studies. Blair et al. [4] stimulated CD19^+^CD24^high^CD38^high^ Bregs with PMA, ionomycin, and CD40 complexes, and they observed the latter's ability to induce the production of IL-10. They found that the proportion of cells decreased and as well as their regulatory function (i.e., the inhibition of the production of proinflammatory cytokines by T cells) [4]. Wang et al. [26] described the number of IL-10^+^ cells and the number of CD19^+^CD5^+^CD1d^+^ cells in patients with new-onset SLE. The number of these cells recovered after administration of immunosuppressive therapy. Isolated PBMC were stimulated with lipopolysaccharide (LPS). Therefore, the uses of different stimulators, as well as the differences in the production of IL-10 of these cell populations, have resulted in differences in research results. In addition, different disease activity levels of the study subjects may also affect the outcome. For example, Sieber et al. [25] found that, in patients with severe SLE, the body's response to TLR9 stimulation was weakened. Although there is currently debate about the definition of the Bregs phenotype, data from the study [23] indicate a decrease in the percentage of IL-10^+^ B cells after stimulation. The proportion of renal pathological IL-10^+^ B cells in patients with lupus nephritis (LN) was significantly lower than that in healthy controls. The proportion of patients with IL-10^+^ B cells was inversely correlated with the daily dose of prednisolone. This suggests that immunosuppressive therapy may affect immune regulation. The study [23] concluded that the ratio of IL-10^+^ B cells can balance the pathogenic and negative regulatory functions of B cells. A decrease in IL-10 secretion is indicative of a decrease in the body's negative immunomodulatory capacity, leading to B cell activation and progression of lupus nephritis.

Multiple studies [27, 28] have confirmed that IL-10 mRNA and protein expression levels were increased in SLE patients. Moreover, almost all studies have tested serum IL-10 concentrations, suggesting that IL-10 is associated with SLE disease activity. The expression levels of IL-10 vary depending on race, age, stimulator, and cell type [29]. The production of IL-10 depends on its gene and protein expression. TNF and IFN regulate IL-10 expression, and their levels can affect IL-10 activity [29]. The expression of serum TNF-*α* and IL-10 was significantly increased in SLE patients in India [30].

Our study [31] found that compared with healthy people, CD19^+^CD24^high^CD38^high^ Bregs and IL-10 were upregulated; IL-10 receptor was downregulated in peripheral blood of SLE patients. The high expression of CD19^+^CD24^high^CD38^high^ Bregs and IL-10 may compensate for the low expression of IL-10 receptor.

IL-10 is both an executive molecule of inhibitory function in Bregs and a promoter of plasma cell differentiation, a contradiction that is particularly pronounced in human SLE disease. For this contradiction, our understanding is that IL-10 plays a leading role in the immunosuppression of normal expression, maintaining the body's immune balance. However, for active autoimmune diseases such as SLE, due to the significant increase in IL-10 expression, its role in promoting plasma cell differentiation is dominant, which in turn promotes immune disorders. This seemingly contradictory two-way immunomodulatory function of IL-10 may be related to its expression in vivo.

Bregs participate in human SLE mainly but not only through IL-10. However, IL-35 [32] and TGF-*β* [31, 33] also play key roles in the pathogenesis of SLE. Study [34] found serum IL-35 levels were lower in active SLE compared to inactive disease and inversely correlated with SLEDAI-2K [34]. Furthermore, serum IL-35 levels were lower in patients with LN compared to SLE patients without LN [34]. Serum IL-35 levels were found decreased in patients with SLE [35] and negatively correlated with the disease activity index. Importantly, treatment with methylprednisolone at a dose 0.8 mg/kg/day restored the reduced IL -35 serum levels within few days [35]. In another study, the biological effects of IL-35 appeared to be reduced [36]. Plasma IL-35 levels were found to be elevated in severe SLE patients but did not exhibit correlation with disease activity [36]. Messenger RNA (mRNA) for p35 and EBI3 were also increased in PBMCs from patients with severe SLE, but IL-35 receptor gp130 expression in CD4 + Th cells was low and inversely correlated with disease activity. The findings of the latter study suggest that the elevated IL-35 levels in severe SLE due to reduced expression of IL-35 receptor were inadequate to expand Tregs [37].

SLE-patients had a significant decreased percentage of Granzyme B+ B-cells in particular SLE-patients with active disease and with lupus nephritis. The data [38] support the hypothesis that the regulatory function of B-cells via Granzyme B is reduced in active disease and in patients with lupus nephritis. The lack of regulation may lead to uncontrolled production of high affinity autoantibodies, formation of immune complexes and subsequently to the initiation and perpetuation of SLE disease [38]. This suggests a pivotal role of Granzyme B for the development of renal involvement. The observation that the secretion is strongly dependent on IL-21 unravels potentially.

Data [26] indicate that the imbalance of IL-21+ follicular helper T cells- (TFH-) like CD27 high and IL-10+ B cells may be associated with the pathogenesis of SLE, and levels of serum IL-21 and IL-10 may be valuable for evaluating disease activity in SLE.

## 4. IL-10, Its Receptor Gene Polymorphisms, and Susceptibility to SLE

In addition, several studies have focused on the susceptibility of IL-10 gene SNPs to SLE susceptibility. Chong et al. [39] reported that the IL-10 gene has multiple SNPs involved in SLE susceptibility: -3575T/A, -2849G/A, -2763C/A, -1082A/G, -819T/C, and -592A/C. Wang et al. [40] found that IL-10-1082G/A was significantly associated with SLE in Asian populations but was not significantly associated in Caucasians. This suggests that the IL-10-1082G/A gene polymorphism is an ethnically specific genetic factor that increases susceptibility to SLE. Another study [41] found that the IL-10 receptor rs2834167 (A/G) polymorphism is associated with SLE susceptibility in a Chinese population. Song et al. [42] showed that the IL-10-1082G/A gene polymorphism is associated with European SLE, and IL-10-819T/C is associated with Asian SLE. Rianthavorn et al. [29] found that the -819 CC and -592 CC genotypes can amplify the susceptibility of juvenile SLE. Recent research found that the IL-10 rs3024498 polymorphism might contribute to SLE susceptibility and several clinical phenotypes [43]. Many factors, such as limited sample size and differences in research methods, can lead to a diversity in research results; thus, these results still need further verification.

## 5. Targeted Therapy Based on Bregs and IL-10/IL-10R

IL-10 antagonists are effective in the treatment of SLE [44]. B cells can be selectively targeted for depletion either via direct B cell molecules such as CD19, CD20, and CD22, or by inhibition of B cell survival factors such as B lymphocyte stimulators, a proliferation-inducing ligand, or their receptors [45]. Recent research indicated that the inhibition of B cell metabolism mediated by two synergistic cytokines, TGF-*β* and IL-10, contributes to the induction of immune tolerance and could be a new therapeutic strategy for autoimmune diseases such as SLE [46]. The proportion of CD5 B cells increased and CD27 memory B cells decreased 12 months after treatment with Rituximab and Dexamethasone compared to the baseline [47], with an inverse correlation between platelet numbers and proportion of CD27^+^ B cells. Both treatment regimens normalized the frequencies of cytokine-producing B cells. The additional increase in CD5^+^B cells after Rituximab and Dexamethasone is compatible with the induction of Bregs.

Costimulatory signals initiate and sustain T-cell responses. Abnormal costimulatory signals also contribute to the pathogenesis and progress of inflammation and autoimmune diseases [48]. Belimumab is a neutralizing monoclonal antibody specific to bind-ing BLyS, which reduces peripheral levels of B cells, and it is an approved therapeutic for SLE [49]. Atacicept inhibits B-cell activation by specifically binding and neutralizing both BLyS and APRIL and thereby reduces B-lymphocyte and Ig levels in lupus patients [50].

Therefore, with the advancement of targeted research on IL-10, IL-10R, and costimulatory signals, diagnostic methods for modulating cytokines are now emerging.

However, as a new target for immunotherapy, Bregs also need to be concerned with the following areas: (1) identifying the definitive markers and specific transcription factors of Bregs and effector B cells is necessary for the development of new B cell-targeted therapeutic strategies; (2) after administration of in vitro cultured Bregs for immunotherapy, the phenotype and biological characteristics of Bregs may change in the body; (3) Bregs are from different stages of B cells in their differentiation and development, but not in the terminal stage. Bregs administered to the body may undergo environmental stimulation and redifferentiate into effector B cells, which may result in adverse effects; (4) a variety of signaling pathways and cytokines can affect the expansion of Bregs. It is not clear whether the biological characteristics of Bregs would change after stimulated or regulated by different signals or immune responses; (5) if the amount of Bregs administered to the body is too little, the clinical treatment effect may be inefficient. However, treatment with excessive numbers of Bregs may excessively inhibit the body's immune response, which can greatly increase the risk of tumor development and infectious diseases. Thus, the number of Bregs used in immunotherapy is important and must be in accordance particular clinical standards or a consensus [51].

## 6. Conclusion and Future Perspectives

In conclusion, the important roles of B cell regulatory elements in maintaining immunotolerance and in controlling and suppressing the inflammatory response have been confirmed in many independent studies. However, the available data are still limited for the proper delineation of the exact role of Bregs in disease pathogenesis. Some questions still need to be answered, such as what the clear phenotypes and characteristic markers of Bregs are, whether different subsets of Bregs participate in immune regulation in different ways or not, and how treatment through targeting Bregs can be made safe and efficient. Therefore, further studies about the role of the Bregs in the inflammatory response will improve our understanding of the etiology of autoimmune diseases and aid in the development of approaches to the therapeutic modalities employing the use of Bregs.

## Figures and Tables

**Table 1:** Phenotype of human regulatory B cells.

Phenotype	References
CD19^+^CD24^hi^CD27^+^	[3, 6, 7]
CD19^+^CD25^hi^CD86^hi^CD1d^hi^	[5]
CD19^+^CD24^hi^CD38^hi^	[4, 8, 9]
CD19^+^CD38^+^CD1d^+^IgM^+^CD147^+^GrB^+^	[10]
CD19^+^CD5^+^CD1d^hi^	[11, 12]
CD19^+^CD5^+^Foxp3^+^	[13]
CD5^+^CD24^hi^CD38^hi^	[14]
CD19^+^CD25^+^	[15]
